# Long-Term Effectiveness Associated With Fecal Immunochemical Testing for Early-Age Screening

**DOI:** 10.1001/jamaoncol.2025.1433

**Published:** 2025-06-12

**Authors:** Han-Mo Chiu, Sam Li-Sheng Chen, Chiu-Wen Su, Amy Ming-Fang Yen, Wen-Feng Hsu, Chen-Yang Hsu, Ting-Yu Lin, Yi-Chia Lee, Ming-Shiang Wu, Tony Hsiu-Hsi Chen

**Affiliations:** 1 Department of Internal Medicine, National Taiwan University Hospital, Taipei, Taiwan; 2 Department of Internal Medicine, College of Medicine, National Taiwan University, Taipei, Taiwan; 3 School of Oral Hygiene, College of Oral Medicine, Taipei Medical University, Taipei, Taiwan; 4 Daichung Hospital, Miaoli City, Taiwan; 5 Institute of Health Analytics and Statistics, College of Public Health, National Taiwan University, Taipei, Taiwan

## Abstract

**Importance:**

The rising incidence of young-onset colorectal cancer (CRC) has prompted health policymakers to consider lowering the recommended starting age for screening. However, population-based evidence supporting the long-term effectiveness of early-age screening remains limited.

**Objective:**

To evaluate whether initiating fecal immunochemical test (FIT) screening at ages 40 to 49 years, rather than at the currently recommended age of 50 years, reduces CRC incidence and mortality.

**Design, Setting, and Participants:**

This study analyzed a community-based screening cohort of Taiwanese residents aged 40 to 49 years, categorized into 4 subcohorts based on participation in early screening (age 40 to 49 years) and continuation of nationwide regular screening (50 years and older). The cohort was followed up until 2019 to compare CRC incidence and mortality across subcohorts. To mitigate self-selection bias, a delayed screening design and efficient propensity score matching was used, restricting analyses to participants attending regular screening. To validate the findings, an extended nonadherence adjustment was applied to all 4 subcohorts. Data were collected from January 2001 to December 2019, and data were analyzed from January 2021 to December 2024.

**Exposures:**

Biennial FIT screening was initiated for the early screening group at ages 40 to 49 years and for the regular screening group at age 50 years, with follow-up continuing under Taiwan’s national screening program.

**Main Outcomes and Measures:**

Primary outcomes were CRC incidence and mortality rates, reported as cases per 100 000 person-years, with adjusted relative risks (aRRs) comparing early vs regular screening groups.

**Results:**

Of 263 125 included participants, 146 796 (55.8%) were female. A total of 39 315 participated in early and regular screening, and 223 810 participated in regular screening only. The early screening group exhibited lower CRC incidence (26.1 [95% CI, 22.3-29.9] vs 42.6 [95% CI, 40.5-44.7] per 100 000 person-years) and mortality (3.2 [95% CI, 1.9-4.6] vs 7.4 [95% CI, 6.5-8.2] per 100 000 person-years). In propensity score–matched analyses, early screening significantly reduced CRC incidence (aRR, 0.79; 95% CI, 0.67-0.94) and mortality (aRR, 0.61; 95% CI, 0.38-0.98). Findings were consistent in the extended nonadherence adjustment model, showing a 25% reduction in incidence (aRR, 0.75; 95% CI, 0.72-0.77) and a 34% reduction in mortality (aRR, 0.66; 95% CI, 0.62-0.71).

**Conclusions and Relevance:**

This study found that initiating FIT screening at age 40 to 49 years was associated with further reduction in CRC mortality and incidence compared with starting screening at age 50 years. These results provide strong empirical support for lowering the CRC screening age, with substantial public health implications.

## Key Points

**Question:**

Can screening for colorectal cancer (CRC) at an earlier age effectively further reduce CRC incidence and mortality compared with usual screening in individuals aged 50 to 75 years?

**Findings:**

In this cohort study including 263 125, fecal immunochemical test (FIT) screening initiated at age 40 to 49 years followed by screening after age 50 years was associated with a 39% and 21% reduction in CRC mortality and incidence, respectively, compared with starting screening at age 50 years.

**Meaning:**

Early FIT screening may improve the effectiveness of existing screening programs starting at age 50 years, supporting the recommendation to lower the age of CRC screening initiation.

## Teaser

This cohort study evaluates whether initiating fecal immunochemical test screening at age 40 to 49 years, rather than at the currently recommended age of 50 years, reduces colorectal cancer incidence and mortality.

## Introduction

Many attempts have been made to establish population-based screening programs for colorectal cancer (CRC), the third most common cancer worldwide,^1,2^ leveraging techniques such as colonoscopy or stool-based testing.^3^ As a result, CRC incidence and mortality have declined following the implementation of screening guidelines^4^ for individuals aged 50 to 75 years over the past 2 decades.

In contrast, CRC incidence and mortality rates have risen in recent years among younger adults, with the most significant increase observed in the age 40 to 49 years group.^5,6,7,8,9^ In response to this trend, several organizations have recently revised their recommendations, lowering the recommended screening initiation age from 50 years to 45 years.^10,11^ However, these recommendations were based primarily on modeling studies extrapolated from previous screening experience in the age 50 to 75 years group, rather than on empirical data from randomized trials or cohort studies specifically involving adults aged 40 to 49 years.^12,13^

A recent observational study has provided supportive evidence for initiating fecal immunochemical test (FIT) screening at an earlier age.^14^ In addition to this short-term evidence, validating the long-term effectiveness of early-age screening is essential for health policymakers.

The current study aimed to investigate whether initiating FIT screening between ages 40 and 49 years would be more effective in reducing CRC incidence and mortality than starting at age 50 years.

## Methods

### Community-Based Screening Cohort Aged 40 to 49 Years

**Figure 1.**
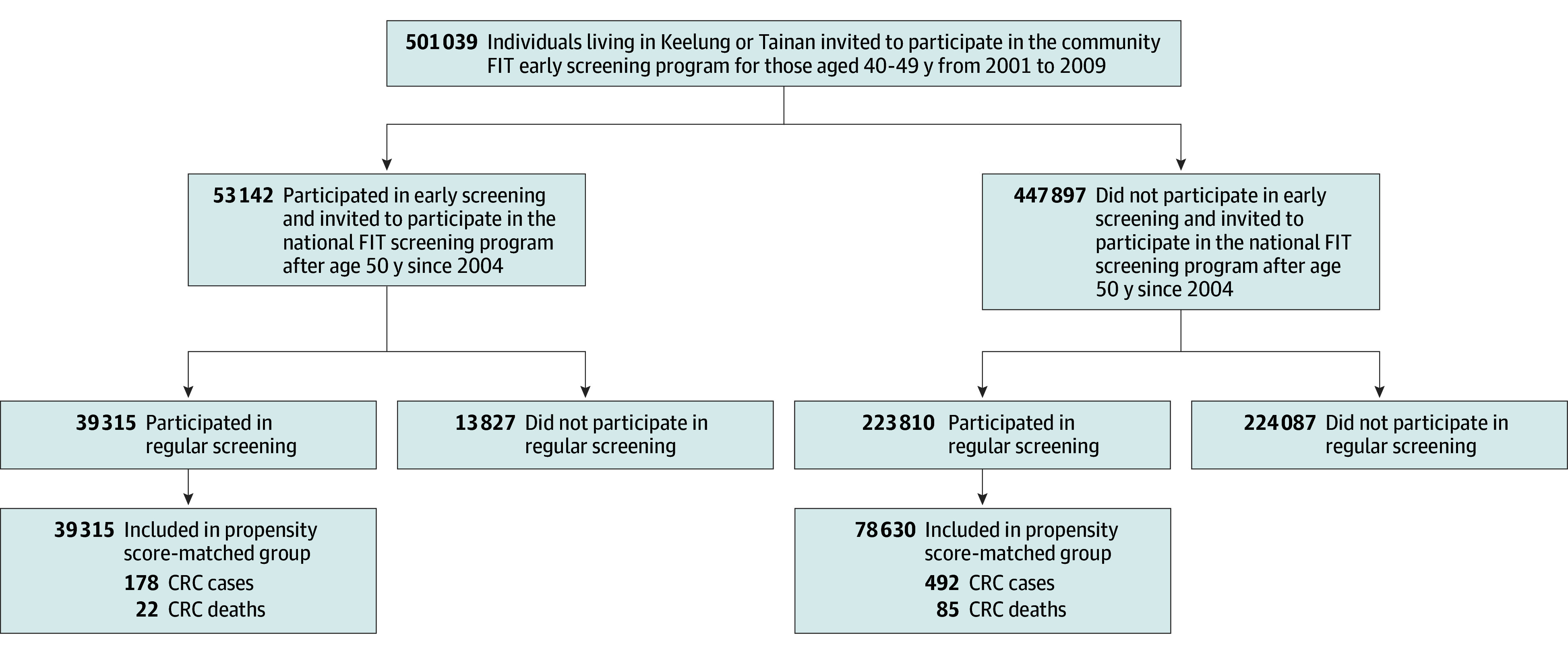
Study Flowchart Study flow showing the delay screen design for deriving the early screening group and the regular screening group from a community-based screening cohort and the corresponding propensity score–matched cohort. CRC indicates colorectal cancer; FIT, fecal immunochemical test.

The study cohort consisted of community members aged 40 to 49 years, identified from Taiwan’s 2 city population registers: Tainan in the south and Keelung in the north (Figure 1). These individuals were recruited before Taiwan’s nationwide CRC screening program for those aged 50 to 69 years. Before this national program, residents aged 40 to 49 years were invited to undergo CRC screening within a broader community-based multiple screening initiative in these 2 municipalities—Keelung since 2001 and Tainan since 2003. Detailed program information is available elsewhere.^15^ This initiative incorporated CRC screening for individuals 40 years and older, using the FIT as the primary tool. This study was approved by the Institutional Review Board of Taipei Medical University. All participants provided written informed consent. This study followed the Strengthening the Reporting of Observational Studies in Epidemiology (STROBE) reporting guideline.

With the launch of Taiwan’s nationwide biennial CRC screening program in 2004 for individuals aged 50 to 69 years, those who had participated in the earlier community-based screening were invited to enroll in the regular screening regimen at age 50 years.^16,17^ This transition enabled an investigation of the long-term impact of early FIT screening (at age 40 to 49 years) compared with screening initiated at age 50 years.

This cohort provided a natural setting for assessing the effectiveness of early FIT screening. Figure 1 presents the study flowchart. Between 2001 and 2009, 501 039 adults aged 40 to 49 years were invited to community-based CRC screening in these cities. Of these, 447 897 did not undergo FIT screening before age 50 years, while 53 142 did. Participants were tracked to determine subsequent participation in the nationwide CRC screening program.

Figure 1 categorizes participants into 4 subcohorts based on early FIT screening at age 40 to 49 years and later regular CRC screening at 50 years and older: 39 315 who underwent early and regular screening, 13 827 who underwent early screening only, 223 810 who underwent regular screening only, and 224 087 who underwent no regular screening. Five-year age-specific CRC incidence and mortality rates were compared across these subcohorts to evaluate long-term benefits.

To address potential biases from self-selection in both early and regular screening, an extended nonadherence adjustment was applied to assess the long-term effectiveness of early-age FIT screening.

### Delayed Screen Design and Propensity Score Matching Method

In addition to analyzing the 4 subcohorts from the age 40 to 49 years group, a delayed screen design was used.^18^ This design focused on 2 groups: those who underwent early and regular screening, representing participants who started FIT screening at age 40 to 49 years and continued in the regular program at 50 years and older and those who underwent regular screening only, representing those who delayed screening until age 50 years. These groups were used for a propensity score–matched analysis.

This approach reduced self-selection bias by restricting analysis to individuals participating in the regular screening program. Propensity score matching then adjusted for self-selection bias in early screening. Given differences in sex, age, and family history of CRC (eTable 1 in Supplement 1), approximately 1:2 matching was applied between the early and regular screening groups to ensure comparability. Figure 1 illustrates the propensity score matching process. Further methodological details can be found in eAppendix 1 and eTables 2 and 3 in Supplement 1.

To ascertain CRC incidence and mortality, the study cohort was linked to the Taiwan Cancer Registry (98.4% completion rate)^19^ and the Taiwan Mortality Registry. Follow-up continued until the end of 2019 or the occurrence of CRC diagnosis or CRC-related mortality. Deaths from other causes were accounted for using a random censorship approach, and person-years of follow-up were calculated.

### FIT

Both the community-based and nationwide screening programs used biennial FIT screening with a quantitative FIT kit (OC-SENSOR; Eiken Chemical), with a positive cutoff of 20 μg hemoglobin per gram of feces. During the community-based program, FIT results were recorded in local health bureau databases. After transitioning to the regular screening program, results were stored in the central government database. Participants were notified of results via mail and phone calls, and those with positive results were scheduled for colonoscopy within 6 months.

### Invasive CRCs by Detection Mode

Incident CRC cases were classified by detection mode. Cancer staging followed the *American Joint Committee on Cancer* (*AJCC*) *Staging Manual*, *sixth edition*, to *AJCC Staging Manual*, *eighth edition*. The primary outcome was incident CRC, encompassing cases diagnosed after the initial screening. These included cancers detected in subsequent screening rounds, interval cancers occurring between negative FIT results, cancers in individuals who did not undergo colonoscopy after a positive FIT result, and CRC diagnosed following an initially negative colonoscopy.

### Statistical Analysis

CRC incidence and mortality rates were calculated as cases and deaths per total person-years. Person-years were determined from study entry to CRC diagnosis, death (competing causes included), or study end point (December 31, 2019).

To assess the impact of early and regular screening, incidence and mortality rates were reported by recruitment age (age 40 to 44 years and age 45 to 49 years) to ensure fair comparisons across subcohorts with equivalent follow-up. Five-year age-stratified rates at diagnosis were also examined to determine the period of greatest benefit for early screening, accounting for lead time and length bias.

Cumulative CRC incidence and mortality rates were reported per 100 000 person-years. Comparisons were conducted for screened vs nonscreened individuals aged 40 to 49 years, the 4 subcohorts, and the propensity score–matched early and regular creening groups.

The long-term effectiveness of early screening was evaluated using a Poisson multivariable regression model on the matched cohort, adjusting for regular screening rounds attended after age 50 years. To confirm robustness, additional Poisson regression models were applied: (1) adjusting for continuous propensity score, (2) adjusting for quintile propensity score, and (3) a fully adjusted model incorporating age, sex, family history of CRC, and number of regular screenings. An extended nonadherence adjustment, based on Duffy’s self-selection bias adjustment approach,^20^ was applied to approximate an intention-to-treat analysis. All analyses were conducted using SAS version 9.4 (SAS Institute).

## Results

### Basic Characteristics Findings

Of 263 125 included participants, 146 796 (55.8%) were female. A total of 39 315 participated in early and regular screening, and 223 810 participated in regular screening only. Table 1 summarizes the general and individual characteristics related to CRC incidence and mortality in the early and regular screening groups used in the delayed screen design. The early screening group had a lower CRC incidence rate compared with the regular screening group in the overall cohort (26.1 [95% CI, 22.3-29.9] vs 42.6 [95% CI, 40.5-44.7] per 100 000 person-years) as well as across each stratum of risk factors. The mean (SD) follow-up period for the 2 groups was 17.4 (1.3) and 17.0 (1.1) years, respectively. Similar findings were observed for CRC mortality. Given a mean (SD) follow-up duration of 17.4 (1.3) and 17.0 (1.0) years, the mortality rate was 3.2 (95% CI, 1.9-4.6) per 100 000 person-years for the early screening group and 7.4 (95% CI, 6.5-8.2) per 100 000 person-years for the regular screening group. Higher CRC incidence was observed with advancing age, male sex, family history of CRC, and infrequent uptake of regular screening. Similar findings were noted for CRC mortality.

**Table 1.** Overall and Personal Attributes Associated With Colorectal Cancer (CRC) Incidence and Mortality in the Early Screening and the Regular Screening Group

Characteristic	Early screening group				Regular screening group			
	Participants, No.	CRC, No.	Person-years at risk	Incidence per 100 000 person-years	Participants, No.	CRC, No.	Person-years at risk	Incidence per 100 000 person-years
**CRC incidence**								
Overall	39 315	178	682 112	26.1	223 810	1620	3 803 860	42.6
Sex								
Male	13 026	72	226 082	31.8	103 303	902	1 759 130	51.3
Female	26 289	106	456 030	23.2	120 507	718	2 044 730	35.1
Recruit age, y								
40-44	32 484	134	557 187	24.0	155 486	924	2 614 154	35.3
45-49	6831	44	124 925	35.2	68 324	696	1 189 706	58.5
Family history^a^								
Yes	2193	12	38 199	31.4	11 213	108	190 476	56.7
No	37 122	166	643 913	25.8	212 597	1512	3 613 384	41.8
Screening rounds with uptake after age 50 y								
1	13 530	88	225 718	39	110 058	1017	1 840 658	55.3
≥2	25 785	90	456 394	19.7	113 752	603	1 963 202	30.7
**CRC death**								
Overall	39 315	22	682 777	3.2	223 810	281	3 810 150	7.4
Sex								
Male	13 026	5	226 348	2.2	103 303	156	1 762 644	8.9
Female	26 289	17	456 429	3.7	120 507	125	2 047 506	6.1
Recruit age, y								
40-44	32 484	14	557 652	2.5	155 486	167	2 617 432	6.4
45-49	6831	8	125 125	6.4	68 324	114	1 192 718	9.6
Family history^a^								
Yes	2193	1	38 255	2.6	11 213	22	190 865	11.5
No	37 122	21	644 522	3.3	212 597	259	3 619 286	7.2
Screening rounds with uptake after age 50, y								
1	13 530	14	226 106	6.2	110 058	204	1 844 964	11.1
≥2	25 785	8	456 671	1.8	113 752	77	1 965 186	3.9

^a^ Only individuals with CRC in their first-degree relatives were considered positive for family history of CRC in this study.

Descriptive results for the 4 subcohorts are detailed in eAppendix 3 in Supplement 1, along with eTable 1 in Supplement 1, which includes demographic characteristics, family history of CRC, and detailed screening histories before and after age 50 years. eTable 4 in Supplement 1 presents the ranking of CRC incidence rates associated with early and regular screening statuses. The trend was consistently observed across all risk factor strata.

As described in the methodology, eTable 5 in Supplement 1 provides detailed incidence and mortality rates by 5-year age groups at diagnosis, stratified by early and regular screening statuses. The results indicate that the group participating in early and regular screening, which initiated screening before age 50 years, had lower CRC incidence and mortality compared with those participating in regular screening only. This difference was particularly evident in the 3 key age groups—age 50 to 54 years, 55 to 59 years, and 60 to 64 years—who derived the greatest benefit from early-age screening during follow-up (approximately 10 to 15 years after recruitment at age 40 to 49 years), while accounting for lead time and length bias at initial screening due to the long and heterogeneous natural history of colorectal neoplasia.

It is important to note that the very low CRC incidence observed in the age 40 to 44 years and age 45 to 49 years groups is partially attributable to self-selection and partially to the lead time window required for incident CRC development. Additionally, the relatively sparse number of CRC cases among individuals aged 65 to 69 years, compared with those aged 50 to 64 years, is likely due to insufficient follow-up time.

eTable 6 in Supplement 1 provides detailed results on the distribution of *AJCC* stages across the 4 subcohorts. eTable 7 in Supplement 1 presents the results of FIT positivity rates, adenoma detection rates, and advanced adenoma detection rates. The descriptions are detailed in eAppendix 3 in Supplement 1.

### Cumulative Incidence and Mortality Comparisons

**Figure 2.**
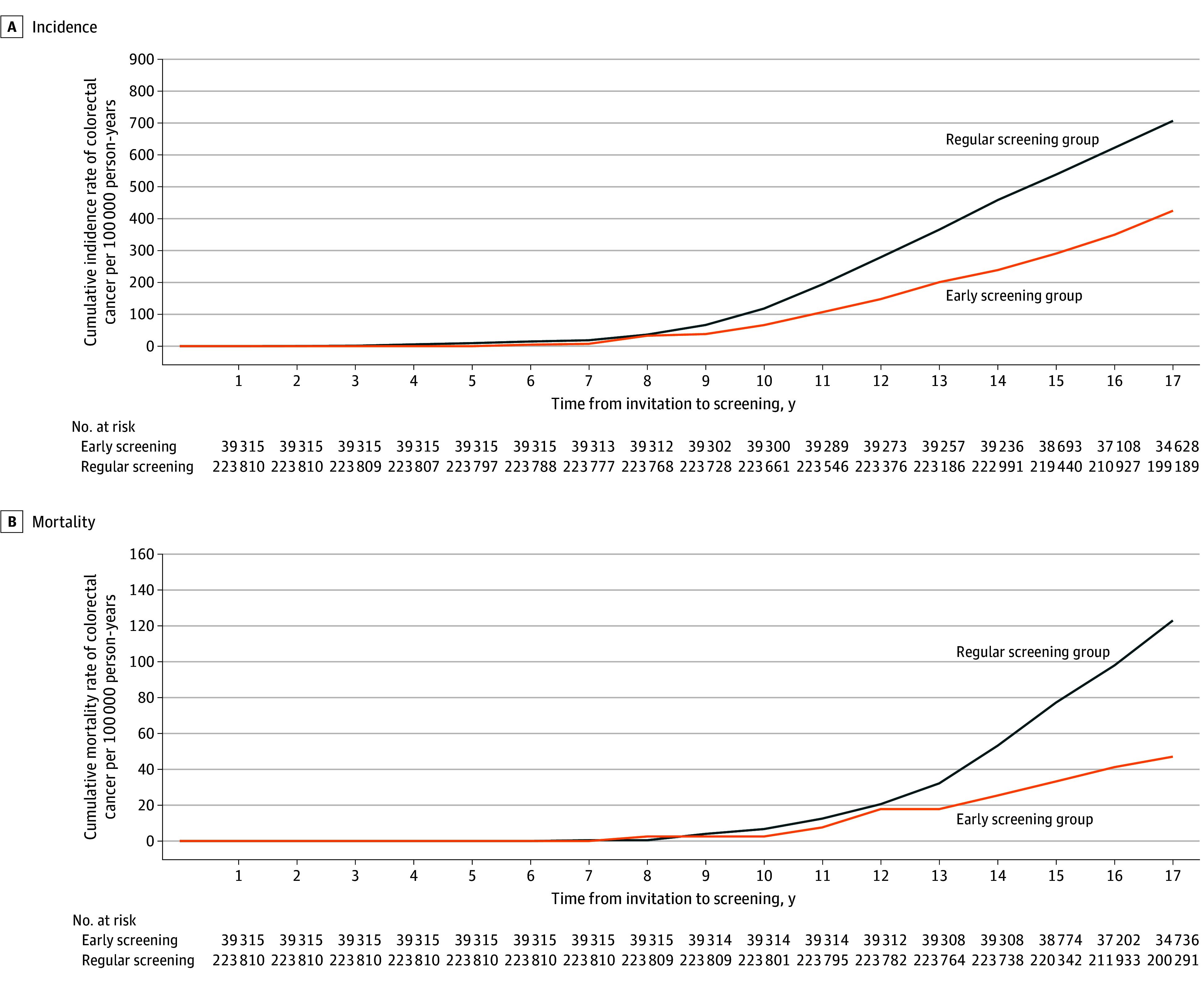
Cumulative Risk of Colorectal Cancer Between the Early and the Regular Screening Groups

Figure 2 demonstrates the consistent long-term benefits of early screening in reducing cumulative CRC incidence and mortality. Similar findings were observed for both male and female participants (eFigures 1 and 2 in Supplement 1).

When comparing the screened group (combining those who participated in early and regular screening and those who participated in early screening only) with the unscreened group (combining those who participated in regular screening only and no regular screening) among individuals aged 40 to 49 years, eFigure 3 in Supplement 1 shows that the cumulative incidence and mortality curves for the screened group vs the unscreened group aged 40 to 49 years were associated with parallel lines of increasing incidence, and the screened group remained higher than the nonscreened group.

eFigure 4 in Supplement 1 displays cumulative incidence and mortality by age at diagnosis from 40 to 69 years for the 4 subcohort groups shown in Figure 1. Notably, the findings indicate that initiating screening before age 50 years and continuing regular screening resulted in the lowest incidence and mortality, followed by initiating screening at age 50 years, initiating screening before age 50 years but not continuing regular screening, and not initiating regular screening, which exhibited the highest incidence and mortality. The closer findings between the latter 2 groups also highlight the importance of continuing regular screening.

### Results of Propensity Score–Matched Analysis

Table 2 presents the corresponding values from the propensity score–matched cohort. It should be noted that after matching, the rate ratios between the 2 groups were more similar in terms of sex, age, and family history than those shown in Table 1.

**Table 2.** Colorectal Cancer (CRC) Incidence and Mortality of Early Screening Group and Regular Screening Group and Matching Factors Among Propensity Score–Matched Cohort

Characteristic	Propensity score–matched cohort							
	Early screening group				Regular screening group			
	Patients, No.	CRC, No.	Person-years at risk	Incidence per 100 000 person-years	Patients, No.	CRC, No.	Person-years at risk	Incidence per 100 000 person-years
**CRC incidence**								
Overall	39 315	178	682 112	26.1	78 630	492	1 333 622	36.9
Sex								
Male	13 026	72	226 082	31.8	26 052	199	441 959	45.0
Female	26 289	106	456 030	23.2	52 578	293	891 663	32.9
Recruit age, y								
40-44	32 484	134	557 187	24.0	64 968	372	1 094 343	34.0
45-49	6831	44	124 925	35.2	13 662	120	239 278	50.2
Family history^a^								
Yes	2193	12	38 199	31.4	4386	36	74 453	48.4
No	37 122	166	643 913	25.8	74 244	456	1 259 169	36.2
Screening rounds with uptake after age 50 y								
1	13 530	88	225 718	39	39 569	322	660 471	48.8
≥2	25 785	90	456 394	19.7	39 061	170	673 150	25.3
**CRC death**								
Overall	39 315	22	682 777	3.2	78 630	85	1 335 410	6.4
Sex								
Male	13 026	5	226 348	2.2	26 052	37	442 674	8.4
Female	26 289	17	456 429	3.7	52 578	48	892 736	5.4
Recruit age, y								
40-44	32 484	14	557 652	2.5	64 968	64	1 095 617	5.8
45-49	6831	8	125 125	6.4	13 662	21	239 793	8.8
Family history^a^								
Yes	2193	1	38 255	2.6	4386	9	74 571	12.1
No	37 122	21	644 522	3.3	74 244	76	1 260 839	6.0
Screening rounds with uptake after age 50, y								
1	13 530	14	226 106	6.2	39 569	63	661 722	9.5
≥2	25 785	8	456 671	1.8	39 061	22	673 689	3.3

^a^ Only individuals with CRC in their first-degree relatives were considered positive for family history of CRC in this study.

Using the matched cohort, Table 3 shows that individuals who started screening between the ages of 40 and 49 years had significantly lower adjusted relative risks (aRRs) of CRC incidence and CRC mortality, with an aRR of 0.79 (95% CI, 0.67-0.94) and 0.61 (95% CI, 0.38-0.98), respectively, compared with those who started screening after age 50 years. Similar significant findings were observed for the continuous propensity score adjustment, with corresponding figures of 0.77 (95% CI, 0.61-0.91) for CRC incidence and 0.58 (95% CI, 0.38-0.91) for CRC mortality, as well as for the decile propensity score adjustment, with figures of 0.77 (95% CI, 0.65-0.90) and 0.58 (95% CI, 0.37-0.89), respectively (eTable 8 in Supplement 1).

**Table 3.** Efficacy of Early Screening vs Regular Screening for Colorectal Cancer (CRC) Incidence and Mortality Using 3 Kinds of Propensity Score Adjustments^a^

Propensity score adjustment	aRR (95% CI)	
	CRC incidence	CRC mortality
Matching	0.79 (0.67-0.94)	0.61 (0.38-0.98)
Continuous	0.77 (0.66-0.91)	0.58 (0.38-0.91)
Decile	0.77 (0.65-0.90)	0.58 (0.37-0.89)

Abbreviation: aRR, adjusted relative risk.

^a^ All 3 methods also account for number of attending the regular screening (incidence model: aRR, 0.50; 95% CI, 0.43-0.58; mortality model: aRR, 0.31; 95% CI, 0.20-0.47). The results of the counterparts in models with continuous and decile adjustment were similar.

eTable 9 in Supplement 1 confirms these findings using the full model, which incorporated adjustments for all relevant variables, including positive associations with a family history of CRC, male sex, and an earlier birth cohort, as well as an inverse association with undergoing CRC screening 2 or more times after age 50 years.

### Results of the Adjustment for Selection Biases Related to Early and Regular Screening

To achieve an intention-to-treat analysis on the incremental long-term effectiveness of early screening vs regular screening, an extended nonadherence bias adjustment (eAppendix 2 in Supplement 1) was applied to data from the 4 subcohorts. The results showed a statistically significant 25% reduction in CRC incidence (aRR, 0.75; 95% CI, 0.72-0.77) and a 34% reduction in CRC mortality (aRR, 0.66; 95% CI, 0.62-0.71) for those invited to early screening compared with those who were not invited.

### Number of Participants Needed to Screen to Prevent One CRC

According to the propensity score–matched results of the current study, the number of participants needed to screen to prevent 1 additional CRC case was 1548 when screening began at ages 40 to 49 years, which was lower than 2079 when screening began at age 50 years, compared with no screening.

## Discussion

In this population-based cohort study of 501 039 individuals followed up for more than 17 years, initiating FIT screening at ages 40 to 49 years effectively lowered CRC incidence and mortality compared with starting at age 50 years. Fewer FIT screenings were needed to prevent 1 CRC case when screening began at 40 to 49 years rather than after age 50 years. These findings highlight FIT’s effectiveness in this younger age group and support recommendations to lower the screening initiation age. Importantly, the long-term benefits of early screening were consistently observed across multiple study designs and statistical adjustments for self-selection bias.

### Long-Term Benefits of Reducing CRC Incidence and Mortality

The greater long-term impact of earlier screening is likely due to the detection of advanced adenomas and early-stage cancers. While FIT’s effectiveness in reducing CRC mortality is well documented, its role in lowering CRC incidence is less studied.^21,22^ Previous studies have shown that FIT screening for those aged 50 to 69 years reduced CRC mortality and incidence by 21% and 33%, respectively.^23,24^

Given the large population aged 45 to 49 years and their relatively lower neoplasm burden, noninvasive FIT screening presents a strong opportunity to reduce CRC incidence and mortality. eTable 5 in Supplement 1 shows that initiating screening before age 50 years provided the greatest benefit among key age groups (age 50 to 54 years, 55 to 59 years, and 60 to 64 years), as it leveraged the lead time window necessary given CRC’s long natural history. This explains the significantly lower CRC incidence in the early screening group compared with others in the age ranges of 40 to 44 years and 45 to 49 years.

### Rising Trend of Early-Onset CRC

CRC screening traditionally starts at age 50 years due to increasing risk with age. However, early-onset CRC (diagnosed before age 50 years) has become a growing concern.^9^ Rising CRC rates in younger individuals are primarily due to increases in advanced-stage disease, rather than increased screening detection, which is more common in older individuals.^25,26^

Several factors contribute to rising CRC rates in younger populations, including higher calorie intake, processed red meat consumption, sedentary lifestyles, and early-life antibiotic exposure.^27,28,29^ The individuals in this study cohort, born between 1952 and 1966, were among the first to experience rising CRC risk in their 40s.^30^ Our study provides valuable insight into the effectiveness of screening at ages 40 to 49 years compared with starting at age 50 years.

Given Taiwan’s high CRC rates among individuals aged 40 to 49 years, these findings strongly support expanding population screening to younger age groups.^4,24^ Recent guideline updates recommending earlier screening align with these findings.

### Implications for Population-Based FIT Screening in Younger Adults

Expanding population-based FIT screening to younger age groups requires significant investment in resources.^31^ However, studies suggest that beginning screening at age 45 years is effective, although the incremental cost per life-year gained may be higher than in the age 50 to 75 years range.^13,32^ Adults aged 45 to 49 years are in their most productive years, making early screening beneficial for individuals, families, and society. FIT provides a noninvasive, efficient method to increase screening uptake while minimizing resource demands. Our findings show that FIT screening at age 40 to 49 years required fewer tests to prevent 1 CRC case compared with regular screening, reinforcing its cost-effectiveness.

### Credibility of Study Findings

Offering FIT screening to residents aged 40 to 49 years in 2 municipalities was an exploratory initiative preceding Taiwan’s mass screening program for individuals 50 years and older. This provided a natural setting for a delayed screening design, where participation status could change within the same individual, reducing individual-level variance. Since both study groups included individuals attending regular screening after age 50 years, this design minimized selection bias.

An extended nonadherence adjustment method was used to estimate long-term effectiveness while mitigating self-selection bias. This was crucial given the observed 5-year age-specific incidence differences between screened and unscreened individuals in both the early and regular screening subcohorts. The consistency of findings across extended bias adjustments and propensity score–matched methods strengthens the credibility of the study. Adjustments for selection bias accounted for the healthy volunteer effect, using both delayed screening and extended nonadherence methods. These approaches yielded 21% and 25% reductions in CRC incidence and 39% and 34% reductions in CRC mortality, respectively.

### Limitations

This study has limitations. First, when early-age screening was introduced, age- and sex-specific FIT cutoff values had not been established, as later studies demonstrated.^33,34,35^ Further research is needed to determine if risk-stratified cutoffs would enhance screening benefits.^36^ Second, cultural, genetic, dietary, and health care differences may affect CRC screening implementation. Thus, whether early screening policies are generalizable to other populations should be evaluated carefully. However, given our findings and recent studies supporting screening from age 45 years, further international research is warranted to guide global health policies on early CRC screening.

## Conclusions

Initiating screening at ages 40 to 49 years, rather than waiting until age 50 years, significantly lowered CRC incidence and mortality over the long term. This was demonstrated using real-world data from a community-based early screening program that transitioned into a national screening initiative with a delayed screening design. These findings support current recommendations to lower the CRC screening initiation age.

## Notes

Supplement 1. **eAppendix 1.** Propensity Score–Matched Design and Analysis **eAppendix 2.** Extended Noncompliance Biases Adjustment Method **eAppendix 3.** Descriptive Results of 4 Subcohorts Before and After 50 Years of Initiating FIT Screening **eTable 1.** Descriptive Results of Sex, Age, Family History, and the Rounds of the Regular Screen Associated With the Early-Age Screening (40-49) and the Regular Screening (50+) **eTable 2.** Estimated Results of Regression Coefficients of 3 Variables Associated With the Outcome of Attending the Early Screening With the Logistic Regression Model **eTable 3.** Distributions of Sex, Age, and Family History Between the Early and Regular Screening Groups With Respect to the Quintile of Propensity Score **eTable 4.** Risks of Colorectal Cancer and its Related Death Derived From the Community Cohort Aged 40-49 Years With the Delay Screen Design in Taiwan **eTable 5.** CRC Incidence and Mortality Rate of 5-Year Age of Diagnosis Associated With the Statuses of Early Screening (40-49) and Regular Screening (50+) **eTable 6.** Stage and Anatomical Site of Colorectal Cancer Derived From the Community Cohort Aged 40-49 Years With the Delay Screen Design in Taiwan **eTable 7.** Screening Findings in Participants Screened Between the Early and the Regular Screening Groups **eTable 8.** Efficacy of Early Screening vs Regular Screening for Colorectal Cancer Incidence and Mortality Using 3 Propensity Score–Based Adjustments **eTable 9.** Results of the Efficacy of the Early Screening Group vs the Regular Screening Group With the Fully Adjusted Model **eFigure 1.** Cumulative Incidence of Colorectal Cancer in Male and Female Population Between the Early and Regular Screening Groups **eFigure 2.** Cumulative Mortality of Colorectal Cancer in Male and Female Population Between the Early and Regular Screening Groups **eFigure 3.** Cumulative Incidence and Mortality of Colorectal Cancer by the Participation Status of Screening at Ages Between 40 and 49 Years **eFigure 4.** Cumulative Incidence and Mortality of Colorectal Cancer by the Participation Status of Screening by Age

Supplement 2. Data Sharing Statement
