# Supplementary material for: Long-Term Effectiveness Associated With Fecal Immunochemical Testing for Early-Age Screening
Source: JAMA Oncol. 2025 Jun 12;11(8):846–54. doi: 10.1001/jamaoncol.2025.1433 (PMC12163714; doi:10.1001/jamaoncol.2025.1433)
Supplement: Supplement 1. — eAppendix 1. Propensity Score–Matched Design and Analysis eAppendix 2. Extended Noncompliance Biases Adjustment Method eAppendix 3. Descriptive Results of 4 Subcohorts Before and After 50 Years of Initiating FIT Screening eTable 1. Descriptive Results of Sex, Age, Family History, and the Rounds of the Regular Screen Associated With the Early-Age Screening (40-49) and the Regular Screening (50+) eTable 2. Estimated Results of Regression Coefficients of 3 Variables Associated With the Outcome of Attending the Early Screening With the Logistic Regression Model eTable 3. Distributions of Sex, Age, and Family History Between the Early and Regular Screening Groups With Respect to the Quintile of Propensity Score eTable 4. Risks of Colorectal Cancer and its Related Death Derived From the Community Cohort Aged 40-49 Years With the Delay Screen Design in Taiwan eTable 5. CRC Incidence and Mortality Rate of 5-Year Age of Diagnosis Associated With the Statuses of Early Screening (40-49) and Regular Screening (50+) eTable 6. Stage and Anatomical Site of Colorectal Cancer Derived From the Community Cohort Aged 40-49 Years With the Delay Screen Design in Taiwan eTable 7. Screening Findings in Participants Screened Between the Early and the Regular Screening Groups eTable 8. Efficacy of Early Screening vs Regular Screening for Colorectal Cancer Incidence and Mortality Using 3 Propensity Score–Based Adjustments eTable 9. Results of the Efficacy of the Early Screening Group vs the Regular Screening Group With the Fully Adjusted Model eFigure 1. Cumulative Incidence of Colorectal Cancer in Male and Female Population Between the Early and Regular Screening Groups eFigure 2. Cumulative Mortality of Colorectal Cancer in Male and Female Population Between the Early and Regular Screening Groups eFigure 3. Cumulative Incidence and Mortality of Colorectal Cancer by the Participation Status of Screening at Ages Between 40 and 49 Years eFigure 4. Cumulative Incidence and Mortality of Colorect [file jamaoncol-e251433-s001.pdf]

Supplemental Online Content

Chiu HM, Chen SLS, Su CW, et al. Long-term effectiveness associated with fecal immunochemical testing for early-age screening. *JAMA Oncol*. Published online June 12, 2025. doi:10.1001/jamaoncol.2025.1433

eAppendix 1. Propensity Score–Matched Design and Analysis
eAppendix 2. Extended Noncompliance Biases Adjustment Method
eAppendix 3. Descriptive Results of 4 Subcohorts Before and After 50 Years of Initiating FIT Screening
eTable 1. Descriptive Results of Sex, Age, Family History, and the Rounds of the Regular Screen Associated With the Early-Age Screening (40-49) and the Regular Screening (50+)
eTable 2. Estimated Results of Regression Coefficients of 3 Variables Associated With the Outcome of Attending the Early Screening With the Logistic Regression Model
eTable 3. Distributions of Sex, Age, and Family History Between the Early and Regular Screening Groups With Respect to the Quintile of Propensity Score
eTable 4. Risks of Colorectal Cancer and its Related Death Derived From the Community Cohort Aged 40-49 Years With the Delay Screen Design in Taiwan
eTable 5. CRC Incidence and Mortality Rate of 5-Year Age of Diagnosis Associated With the Statuses of Early Screening (40-49) and Regular Screening (50+)
eTable 6. Stage and Anatomical Site of Colorectal Cancer Derived From the Community Cohort Aged 40-49 Years With the Delay Screen Design in Taiwan
eTable 7. Screening Findings in Participants Screened Between the Early and the Regular Screening Groups
eTable 8. Efficacy of Early Screening vs Regular Screening for Colorectal Cancer Incidence and Mortality Using 3 Propensity Score–Based Adjustments
eTable 9. Results of the Efficacy of the Early Screening Group vs the Regular Screening Group With the Fully Adjusted Model
eFigure 1. Cumulative Incidence of Colorectal Cancer in Male and Female Population Between the Early and Regular Screening Groups
eFigure 2. Cumulative Mortality of Colorectal Cancer in Male and Female Population Between the Early and Regular Screening Groups

© 2025 Chiu HM et al. *JAMA Oncology*.

eFigure 3. Cumulative Incidence and Mortality of Colorectal Cancer by the Participation Status of Screening at Ages Between 40 and 49 Years
eFigure 4. Cumulative Incidence and Mortality of Colorectal Cancer by the Participation Status of Screening by Age

This supplementary material has been provided by the authors to give readers additional information about their work.

© 2025 Chiu HM et al. *JAMA Oncology*.

eAppendix 1. Propensity Score–Matched Design and Analysis

As shown in Figure 1, the delayed screening design, while providing a natural setting, is not a randomized controlled trial but an exploratory study. Consequently, there is a likelihood of imbalance between the early-screening and regular-screening groups, as the number of participants in the former is relatively low compared with the latter (39,315 vs. 223,810; see the middle panel of Figure 1). This imbalance could introduce self-selection bias, which is strongly associated with female sex and younger adults aged 40-44 compared with those aged 45-49 and, to a lesser extent, family history (see **Table S1**). This self-selection bias is inherent to multiple community-based service screening programs, described in detail elsewhere (Chen et al., 2004).

To account for this imbalance and self-selection, we applied a propensity score-matching method to balance the early-screening and regular-screening groups in terms of sex, age at entry, and family history. A logistic regression model was constructed to predict the likelihood of attending early screening based on these three variables. The probability of attending early screening was then used to calculate each participant's propensity score.

For each enrolled subject i, the logistic regression equation is formulated as follows:

© 2025 Chiu HM et al. *JAMA Oncology*.

$$\text{Logit}(P(\text{Group}_i \mid \text{Age}_i, \text{Sex}_i, \text{Family history}_i)) = \eta_i \text{ (propensity score)}$$

$$\eta_i = \alpha + \beta_1 \times \text{Age}_i + \beta_2 \times \text{Sex}_i + \beta_3 \times (\text{Family history})_i \qquad \text{(S1)}$$

where Group_i is a binary variable representing whether the individual belongs to the early-screening group (Group=1) or the regular-screening group (Group=0).

Subjects in the early-screening group were matched with those in the regular-screening group based on their logistic regression score (η_i). Matching was performed using a greedy nearest-neighbor matching algorithm (Austin, 2012) with precision up to the fifth decimal point. The early-screening and regular-screening groups were matched at a 1:2 ratio.

The estimated regression coefficients for the logistic regression specified in formula S1 are listed in **Table S2**. The results indicate that age, sex, and family history all have a significant impact on early screening uptake. Females were more likely to attend early screening than males, as reflected by a positive regression coefficient ($P < 0.0001$). Younger adults were also more likely to participate in early screening, with positive estimated regression coefficients for those under 44 years ($P < 0.0001$). Since this is a staggered-entry cohort commencing at age 40, the model accounted for the calendar year of entry when screening individuals. Additionally, individuals with a family history had a higher likelihood of attending early screening ($P = 0.0009$). For the matching process, propensity scores for each recruited subject in the study

© 2025 Chiu HM et al. *JAMA Oncology*.

cohort were calculated using these estimated coefficients and personal attributes. **Table S3** compares the distributions of sex, age, and family history in the early- and regular-screening groups across propensity score quintiles. Within each quintile, homogeneity between the early-screening and regular-screening groups was observed regarding sex, age, and family history of colorectal cancer. These findings suggest that the propensity-score-matching approach effectively balanced the distributions of these key variables between the two groups.

Based on the propensity scores, the 1:2 matching process resulted in 39,315 matched pairs, consisting of 39,315 subjects who attended early screening and 78,630 who attended the regular screening program (see the bottom panel of Figure 1). This matching approach minimized self-selection bias inherent to our non-randomized FIT screening program, enabling an assessment of the effectiveness of early screening compared with regular screening.

In addition to the propensity-score-matching design, we applied two additional adjustment methods: continuous score adjustment and quintile score adjustment. **Table S8** summarizes the results of these three approaches, demonstrating consistent estimates of the efficacy of early screening in reducing colorectal cancer incidence by 21%-23% and mortality by 39%-42% compared with regular screening.

© 2025 Chiu HM et al. *JAMA Oncology*.

References

Austin PC (2014). A comparison of 12 algorithms for matching on the propensity score. Statistics in Medicine, 33(6), 1057-1069.

Chen TH, Chiu YH, Luh DL, et al. Community-based multiple screening model: design, implementation, and analysis of 42,387 participants. Cancer 2004;100(8):1734-43. DOI: 10.1002/cncr.20171.

© 2025 Chiu HM et al. *JAMA Oncology*.

eAppendix 2. Extended Noncompliance Biases Adjustment Method

To achieve an intention-to-treat analysis comparable to a randomized controlled trial design with two arms—the invited group (denoted as I , invitation to early-age screening between ages 40 and 49) and the uninvited group (denoted as $\bar{I}$, delayed screening until regular screening after age 50)—we leveraged data from all four sub-cohorts: early (+) regular (+), early (+) regular (-), early (-) regular (+), and early (-) regular (-), as shown in the middle panel of Figure 1. This contrasts with the propensity-score method, which utilized only two groups, early (+) regular (+) and early (-) regular (+), within the context of the delayed screening design.

The detailed methodology, mathematical formula, and notations are derived as follows. The relative risk (RR) was estimated as the ratio of the risk of developing incident CRC (denoted as C) in the invited group (P(C|I)) to the risk of developing incident CRC in the uninvited group (P(C| $\bar{I}$)) as expressed in the second component of the following formula (S2).

$$\text{Relative Risk } (RR_c)=\frac{P(C|I)}{P(C|\bar{I})}=\frac{P(C_1 \cup C_2|I)}{P(C_1 \cup C_2|\bar{I})}=\frac{P(C_1|I)+P(C_2|I)}{P(C_1|\bar{I})+P(C_2|\bar{I})}$$

$$=\frac{P(C_1|A_1,I)P(A_1|I)+P(C_1|\bar{A}_1,I)P(\bar{A}_1|I)+P(C_2|A_1,I)P(A_1|I)+P(C_2|\bar{A}_1,I)P(\bar{A}_1|I)}{P(C_1|\bar{I})+P(C_2|\bar{I})} \quad \text{(S2)}$$

Because the numerators included both early-age screening (ages 40-49) and regular

© 2025 Chiu HM et al. *JAMA Oncology*.

screening (after age 50), the total number of incident CRC cases (C) (the second component of the right-hand-side of the equation S2) can be divided into two components (the third and fourth components of the right-hand-side of the equation S2) denoted by C_1 and C_2 with the subscripts 1 and 2, representing incident CRC cases from early screening (C_1) and incident CRC cases from regular screening (C_2), respectively.

To adjust for non-compliance biases arising from two screening opportunities offered at two landmark age ranges (40-49 vs. 50+), the fifth component of the right-hand-side of the equation S2 is first decomposed based on participants' early-age screening status. A_1 and $\bar{A}_1$ represent compliance and non-compliance with participation, corresponding to early (+) and early (-), respectively (see Figure 1). Again, the subscript 1 refers to early-age screening. It is important to note that when compliance information (A_1 and $\bar{A}_1$) is known, the invitation (I) provides no additional information regarding the risk of developing incident CRC. In other words, the risk of incident CRC is independent of I when conditional on A. As a result, formula S2 can be simplified to formula S3.

$$= \frac{P(C_1|A_1)P(A_1|I) + P(C_1|\bar{A}_1)P(\bar{A}_1|I) + P(C_2|A_1)P(A_1|I) + P(C_2|\bar{A}_1)P(\bar{A}_1|I)}{P(C_1|\bar{I}) + P(C_2|\bar{I})}$$

(S3)

© 2025 Chiu HM et al. *JAMA Oncology*.

$P(C_1|A_1)$ and $P(C_1|\bar{A}_1)$ represent the risks of incident CRC among screened and non-screened individuals during early-age screening, respectively. $P(A_1|I)$ and $P(\bar{A}_1|I)$ indicate the screening rates for early-age screening and its counterpart. All these values are available from Figure 1, **Table S1**, and the incident CRC section of **Table S4**. The first self-selection bias adjustment for early-age screening participation has been completed.

To further account for the second self-selection bias adjustment, the two latter components in the numerator of formula S3, related to CRC incidence after age 50, can be further decomposed based on compliance with regular screening (A_2) and non-compliance ($\bar{A}_2$), corresponding to the notation of regular (+) and regular (-) (see Figure 1) throughout the text, as shown below.

$$P(C_2|A_1)P(A_1|I)$$
$$= P(C_2|A_2,A_1)P(A_2|A_1)\,P(A_1|I) + P(C_2|\bar{A}_2,A_1)P(\bar{A}_2|A_1)P(A_1|I)$$

, and

$$P(C_2|\bar{A}_1)P(\bar{A}_1|I)$$
$$= P(C_2|A_2,\bar{A}_1)P(A_2|\bar{A}_1)\,P(\bar{A}_1|I) + P(C_2|\bar{A}_2,\bar{A}_1)P(\bar{A}_2|\bar{A}_1)P(\bar{A}_1|I)$$

© 2025 Chiu HM et al. *JAMA Oncology*.

$P(C_2|A_2, A_1)$ represents the risk of developing incident CRC among individuals who attended both early-age screening and regular screening. $P(A_2|A_1)$ denotes the screening rate for regular screening among individuals who previously attended early-age screening. Similar definitions apply to all corresponding counterparts. Again, all these values are available from **Figure 1, Table S1,** and the incident CRC section of **Table S4**. Formula S3 is now expanded into Formula S4.

$$= \frac{\left(\begin{array}{c} P(C_1|A_1)P(A_1|I) + P(C_1|\bar{A}_1)P(\bar{A}_1|I) + \\ P(C_2|A_2,A_1)P(A_2|A_1)\ P(A_1|I) + P(C_2|\bar{A}_2,A_1)P(\bar{A}_2|A_1)P(A_1|I) + \\ P(C_2|A_2,\bar{A}_1)P(A_2|\bar{A}_1)\ P(\bar{A}_1|I) + P(C_2|\bar{A}_2,\bar{A}_1)P(\bar{A}_2|\bar{A}_1)P(\bar{A}_1|I) \end{array}\right)}{P(C_1|\bar{I}) + P(C_2|\bar{I})} \tag{S4}$$

The second self-selection bias adjustment has been completed.

Regarding the denominator of the risks of incident CRC for the uninvited group in formulas S2–S4, both the risks of developing incident CRC during the early-age screening and regular screening periods, $P(C_1|\bar{I})$ and $P(C_2|\bar{I})$, were estimated by constructing counterfactual figures in the absence of screening. To achieve this, we used the entire Taiwanese population as the uninvited group, as nearly all individuals in Taiwan were not offered early-age screening during the period when early-age screening was being studied in the two community-based programs. The population was further stratified by sex and 5-year age bands (40-44, 45-49, …, 60-69).

© 2025 Chiu HM et al. *JAMA Oncology*.

To account for the delayed screening design and the counterfactual group for early-age screening in the two communities, the two estimates were adjusted based on changes in age distribution over time within the cohort and baseline incidence differences between the two community studies (Keelung and Tainan) and the general Taiwanese population. A similar procedure was applied to calculate the relative risk (RR) of CRC mortality:

$$\text{Relative Risk } (\text{RR}_\text{D})=\frac{P(D|I)}{P(D|\bar{I})} \qquad \text{(S5)}$$

The numerator is identical to that of formula S4, except that the event of interest is changed to CRC death (D). The formula for the intention-to-treat analysis assessing the efficacy of early-age screening in reducing CRC mortality is expressed as follows:

$$= \frac{\begin{pmatrix} P(D_1|A_1,I)P(A_1|I) + P(D_1|\bar{A}_1,I)P(\bar{A}_1|I) + \\ P(D_2|A_2,A_1)P(A_2|A_1)\ P(A_1|I) + P(D_2|\bar{A}_2,A_1)P(\bar{A}_2|A_1)P(A_1|I) + \\ P(D_2|A_2,\bar{A}_1)P(A_2|\bar{A}_1)\ P(\bar{A}_1|I) + P(D_2|\bar{A}_2,\bar{A}_1)P(\bar{A}_2|\bar{A}_1)P(\bar{A}_1|I) \end{pmatrix}}{P(D|C_1,\bar{A}_2)P(C_1|\bar{A}_2)P(\bar{A}_2|\bar{I}) + P(D|C,A_2)P(C_2|A_2)P(A_2|\bar{I}) + P(D|C,\bar{A}_2)P(C_2|\bar{A}_2)P(\bar{A}_2|\bar{I})}$$

(S6)

Again, all figures required for numerator estimates are available from **Figure 1**, **Table 1**, and the colorectal cancer mortality section of **Table S4**.

To calculate the denominator, we used formula S4 to estimate CRC incidence risk. We then applied two case-fatality rates from the Taiwan CRC screening program:

© 2025 Chiu HM et al. *JAMA Oncology*.

one for individuals who underwent screening ($P(D|C_2, A_2)$) and one for those who did not ($P(D|C_2, \bar{A}_2)$).

To estimate the CRC mortality rate of the uninvited group in the early-age screening cohort, we applied these case-fatality rates to the expected incident CRC cases among participants (A_2) and non-participants ($\bar{A}_2$) in the regular screening program. The most challenging estimate pertains to $P(D|C_1, \bar{A}_2)$, as the corresponding value for participants in regular screening, $P(D|C_1, A_2)$ is not expected to exist due to the temporal sequence—individuals diagnosed before age 50 (C_1) would not yet have had the opportunity to undergo regular screening (A_2) before dying from CRC.

We assumed that the case-fatality rate for individuals diagnosed between ages 40 and 49 is similar to that of those aged 50 and older. Therefore, the estimate of $P(D|C_1, \bar{A}_2)$ can be approximated by $P(D|C_2, \bar{A}_2)$. Recall that we must set $P(C_1| A_2)=0$ due to the contradictory temporal association between a CRC diagnosis before age 50 (C_1) and participation in regular screening after age 50 (A_2). This implies that the denominator of formula S6 consists of only three components.

The aforementioned procedures incorporate adjustments for non-compliance biases in assessing the long-term effectiveness of early-age screening in reducing CRC mortality.

© 2025 Chiu HM et al. *JAMA Oncology*.

eAppendix 3. Descriptive Results of 4 Subcohorts Before and After 50 Years of Initiating FIT Screening

Table S1 details the demographic and personal characteristics of the study population, including sex, age, family history of colorectal cancer, and the number of screening rounds before and after age 50, stratified by the four sub-cohorts. Notably, females had a higher screening uptake on both screening occasions compared with males. Younger individuals aged 40-44 years were more likely to participate in screening than those aged 45-49 years. The early (+) regular (+) group (5.6%) had a higher proportion of individuals with a family history of colorectal cancer than the early (+) regular (-) group (2.1%) but only slightly higher than the early (-) regular (+) group (5.0%).

The early-screening group (early (+) regular (+)) accounted for the majority of first-round screenings (73%), with only 27% undergoing more than two screening rounds between ages 40 and 49. However, their continued uptake of regular screening beyond age 50 increased to 66%, which was higher than the 51% observed in the regular-screening group (early (-) regular (+)).

Table S4 provides details on CRC cases and person-years at risk, yielding incidence and mortality rates for the four sub-cohorts (early (+) regular (+), early (+) regular (-), early (-) regular (+), and early (-) regular (-)) as displayed in Figure 1.

Table S5 details CRC incidence and mortality by five-year age at diagnosis.

© 2025 Chiu HM et al. *JAMA Oncology*.

Three key age groups—50-54, 55-59, and 60-64—are highlighted to compare incidence and mortality between early screening and regular screening, accounting for lead-time and follow-up duration.

Table S6 presents the distribution of AJCC stages across the four sub-cohorts. The proportion of stage IV cases follows this order: 18.2% early (+) regular (+) < 19.3% early (-) regular (+) < 24.1% early (+) regular (-) < 31.4% early (-) regular (-). Early (+) regular (+) group also detected more proximal CRC cases earlier. Both findings highlighted attending both early and regular screening gained the best benefit.

Table S7 provides results on adenoma and advanced adenoma detection. The lower positivity rate, adenoma detection rate, and advanced adenoma detection rate in the early-screening group compared with the regular-screening group likely reflect both the slower growth rate of adenomas with age, given the long disease natural history of colorectal neoplasia, and the quality of colonoscopy in the early years of this community-based cohort. These factors suggest that there is still room for improving FIT sensitivity and highlight the importance of quality control in colonoscopy when implementing a two-stage screening strategy for individuals younger than 50. This study was approved by the Institutional Review Board of Taipei Medical University, under protocol number TMU-JIRBN No.: 202307023.

© 2025 Chiu HM et al. *JAMA Oncology*.

eTable 1. Descriptive Results of Sex, Age, Family History, and the Rounds of the Regular Screen Associated With the Early-Age Screening (40-49) and the Regular Screening (50+)

	The screened Group (age 40-49) (n=53,142)				The non-screened group (age 40-49) (n=447,897)			
	Early (+) Regular (+)[b] (n=39,315)		Early (+) Regular (-) (n=13,827)		Early (-) Regular (+) (n=223,810)		Early (-) Regular (-) (n=224,087)	
	n	%	n	%	n	%	n	%
**Sex**								
Male	13,026	33.1	6,017	43.5	103,303	46.2	134,619	60.1
Female	26,289	66.9	7,810	56.5	120,507	53.8	89,468	39.9
**Age of Recruitment**								
40-44	32,484	82.6	12,641	91.4	155,486	69.5	184,832	82.5
45-49	6,831	17.4	1,186	8.6	68,324	30.5	39,255	17.5
**Family History[a]**								
Yes	2,193	5.6	300	2.2	11,213	5.0		
No	37,122	94.4	13,527	97.8	212,597	95.0		
**Number of screening rounds participating in the early screening between 40 and 49 years of age**								
1	28,650	72.9	11,538	83.4				
2	7,021	17.9	1,709	12.4				
3	1,777	4.5	359	2.6				

© 2025 Chiu HM et al. *JAMA Oncology*.

4	962	2.4	135	1.0		
>=5	905	2.4	86	0.6		
**Number of screening rounds participating in the screening after 50 years of age**						
1	13,530	34.4			110,058	49.2
2	11,402	29.0			62,083	27.7
3	8,391	21.3			34,803	15.6
4	4,128	10.5			12,736	5.7
>=5	1,864	4.8			4,130	1.9

a: Only individuals with colorectal cancer in their first-degree relatives are considered positive for family history in this study

b: early (+) regular (+), early (+) regular (-), early (-) regular (+), and early (-) regular (-) represent four sub-cohorts including comply with both early screening and regular screening, attend early screen but withdraw from regular screening, did not attend early screening but comply with regular screening, did not attend both early screening and regular screening

© 2025 Chiu HM et al. *JAMA Oncology*.

eTable 2. Estimated Results of Regression Coefficients of 3 Variables Associated With the Outcome of Attending the Early Screening With the Logistic Regression Model

Parameter	Estimate	Standard Error	P-value
**Intercept**	-1.8231	0.013	
**Sex**			
Male	1	-	<0.0001
Female	0.2735	0.00581	
**Age at Entry (Calendar year)**			<0.0001
40 (2001)	0.355	0.0175	
40 (2002)	0.3143	0.0183	
40 (2003)	0.2617	0.0194	
40 (2004)	0.2578	0.0206	
40 (2005)	0.2071	0.0236	
40 (2006)	-0.0673	0.0316	
41 (2001)	0.3493	0.0173	
42 (2001)	0.2988	0.0174	
43 (2001)	0.1378	0.0183	
44 (2001)	-0.0039	0.0193	
45 (2001)	1	-	
46 (2001)	-0.5344	0.0231	
47 (2001)	-1.3234	0.0322	
48 (2001)	0.2546	0.04	
49 (2001)	-0.3589	0.0486	
**Family History** (vs. No)			
Yes	0.0404	0.0122	0.0009

© 2025 Chiu HM et al. *JAMA Oncology*.

eTable 3. Distributions of Sex, Age, and Family History Between the Early and Regular Screening Groups With Respect to the Quintile of Propensity Score

Variable		Quintile of Propensity Score																			
		1				2				3				4				5			
		Early		Regular		Early		Regular		Early		Regular		Early		Regular		Early		Regular	
		n	%	n	%	n	%	n	%	n	%	n	%	n	%	n	%	n	%	n	%
**Sex**	Female	608	16.4	11281	21.2	1701	23.5	12833	26.5	2695	38.0	14151	35.4	10070	100	42556	100	11215	100	39686	100
	Male	3101		41992		5531		35537		4394		25774		0		0		0		0	
**Age**	Mean, (sd)	45.4	(2.7)	44.6	(3.3)	42.0	(3.9)	41.7	(3.9)	40.9	(3.0)	41.0	(3.0)	40.5	(3.5)	40.4	(3.5)	40.5	(1.3)	40.5	(1.3)
**Family history**	Yes	119	3.2	1814	3.4	243	3.4	1828	3.8	575	8.1	3037	7.6	264	2.6	1205	2.8	992	8.8	3329	8.4
	No	3590		51459		6989		46542		6514		36888		9806		41351		10223		36357	

© 2025 Chiu HM et al. *JAMA Oncology*.

eTable 4. Risks of Colorectal Cancer and its Related Death Derived From the Community Cohort Aged 40-49 Years With the Delay Screen Design in Taiwan

	The screened Group (age 40-49) (n=53,142)						The non-screened group (age 40-49) (n=447,897)					
	Early (+) Regular (+) (n=39,315)			Early (+) Regular (-) (n=13,827)			Early (-) Regular (+) (n=223,810)			Early (-) Regular (-) (n=224,087)		
	**Colorectal cancer incidence**											
	CRC, n	PY at risk	Incidence per 10^5 PY	CRC, n	PY at risk	Incidence per 10^5 PY	CRC, n	PY at risk	Incidence per 10^5 PY	CRC, n	PY at risk	Incidence per 10^5 PY
**Overall**	178	682,112	26.1	194	229,001	84.7	1,620	3,803,860	42.6	3,117	3,437,240	90.7
**Sex**												
Male	72	226,082	31.8	75	99,504	75.4	902	1,759,130	51.3	1,907	2,026,978	94.1
Female	106	456,030	23.2	119	129,497	91.9	718	2,044,730	35.1	1,210	1,410,262	85.8
**Age of Recruitment**												
40-44	134	557,187	24.0	161	207,538	77.6	924	2,614,154	35.3	2,162	2,832,151	76.3
45-49	44	124,925	35.2	33	21,463	153.8	696	1,189,706	58.5	955	605,089	157.8
**Family History**												
Yes	12	38,199	31.4	8	4,993	160.2	108	190,476	56.7	-	-	-
No	166	643,913	25.8	186	224,008	83	1512	3,613,384	41.8	-	-	-
**Number of screening rounds participated in after 50**												
1	88	225,718	39	-	-	-	1,017	1,840,658	55.3	-	-	-
2 or more	90	456,394	19.7	-	-	-	603	1,963,202	30.7	-	-	-
	**Colorectal cancer death**											

© 2025 Chiu HM et al. *JAMA Oncology*.

	CRC death, n	PY at risk	Mortality per 10^5 PY	CRC death, n	PY at risk	Mortality per 10^5 PY	CRC death, n	PY at risk	Mortality per 10^5 PY	CRC death, n	PY at risk	Mortality per 10^5 PY
**Overall**	22	682,777	3.2	57	230,352	24.7	281	3,810,150	7.4	1,019	3,428,934	29.7
**Sex**												
Male	5	226,348	2.2	20	100,030	20.0	156	1,762,644	8.9	608	2,022,672	30.1
Female	17	456,429	3.7	37	130,322	28.4	125	2,047,506	6.1	411	1,406,262	29.2
**Age of Recruitment**												
40-44	14	557,652	2.5	46	208,656	22.0	167	2,617,432	6.4	676	2,827,290	23.9
45-49	8	125,125	6.4	11	21,696	50.7	114	1,192,718	9.6	343	601,644	57.0
**Family History**												
Yes	1	38,255	2.6	3	5,055	59.3	22	190,865	11.5	-	-	-
No	21	644,522	3.3	54	225,296	24.0	259	3,619,286	7.2	-	-	-
**Number of screening rounds participated in after 50**												
1	14	226,106	6.2	-	-	-	204	1,844,964	11.1	-	-	-
2 or more	8	456,671	1.8	-	-	-	77	1,965,186	3.9	-	-	-

CRC: Colorectal cancer; PY: person-year

© 2025 Chiu HM et al. *JAMA Oncology*.

eTable 5. CRC Incidence and Mortality Rate of 5-Year Age of Diagnosis Associated With the Statuses of Early Screening (40-49) and Regular Screening (50+)

	**The screened Group (age 40-49) (n=53,142)**						**The non-screened group (age 40-49) (n=447,897)**					
	Early (+) Regular (+) (n=39,315)			Early (+) Regular (-) (n=13,827)			Early (-) Regular (+) (n=223,810)			Early (-) Regular (-) (n=224,087)		
	CRC, n	PY at risk	Rate per 10^5 PY	CRC, n	PY at risk	Rate per 10^5 PY	CRC, n	PY at risk	Rate per 10^5 PY	CRC, n	PY at risk	Rate per 10^5 PY
**CRC Incidence**												
**Overall**	178	682,112	26.1	194	229,001	84.7	1,620	3,803,860	42.6	3,117	3,437,240	90.7
**Age of diagnosis**												
40-44	0	101,117	-	28	50,071	55.9	0	426,465	-	249	654,488	38.0
45-49	4	182,647	2.2	68	66,572	102.1	19	910,951	2.1	722	970,567	74.4
50-54	61	195,349	31.2	59	66,639	88.5	455	1,111,552	40.9	977	1,016,491	96.1
55-59	79	147,597	53.5	32	35,617	89.8	690	883,564	78.1	774	573,606	134.9
60-64	30	51,877	57.8	7	9,518	73.5	421	433,466	97.1	374	208,734	179.2
65-69	4	3,524	113.5	0	584	-	35	37,862	92.4	21	13,354	157.3
**CRC Mortality**										-	-	-
**Overall**	22	682,777	3.2	57	230,352	24.7	281	3,810,150	7.4	1,019	3,428,934	29.7
**Age of diagnosis**										-	-	-
40-44	0	101,079	-	2	50,098	4.0	0	426,473	-	48	654,491	-
45-49	0	182,603	-	14	66,776	21.0	0	910,973	-	176	970,385	2.2

© 2025 Chiu HM et al. *JAMA Oncology*.

50-54	4	195,510	2.0	21	67,133	31.3	40	1,112,326	3.6	344	1,015,062	31.2
55-59	10	147,968	6.8	14	36,066	38.8	109	886,120	12.3	289	570,230	53.5
60-64	7	52,078	13.4	6	9,687	61.9	120	436,057	27.5	147	205,773	57.8
65-69	1	3,540	28.2	0	591	-	12	38,201	31.4	15	12,993	113.5

© 2025 Chiu HM et al. *JAMA Oncology*.

eTable 6. Stage and Anatomical Site of Colorectal Cancer Derived From the Community Cohort Aged 40-49 Years With the Delay Screen Design in Taiwan

	The screened Group (age 40-49) (n=372)				The non-screened group (age 40-49) (n=4,737)			
	Early (+) Regular (+) (n=178)		Early (+) Regular (-) (n=194)		Early (-) Regular (+) (n=1,620)		Early (-) Regular (-) (n=3,117)	
	n	(%)	n	(%)	n	(%)	n	(%)
AJCC stage								
I	46	27.9	36	22.8	440	28.6	386	13.8
II	25	15.2	24	15.2	270	17.6	550	19.7
III	64	38.8	60	38.0	530	34.5	978	35.0
IV	30	18.2	38	24.1	297	19.3	877	31.4
Missing	13	-	36	-	83	-	326	-
Anatomical site								
Distal	121	68.0	147	77.0	1,206	74.4	2,408	77.5
Proximal	57	32.0	44	23.0	414	25.6	699	22.5

© 2025 Chiu HM et al. *JAMA Oncology*.

eTable 7. Screening Findings in Participants Screened Between the Early and the Regular Screening Groups

	**Early-screening group** Early (+) Regular (+) (n=39,315)	**Regular-screening group** Early (-) Regular (+) (n=223,810)
**Screening finding at the ages between 40 and 49**		
FIT positive, n (%)	1,904 (4.8)	-
All Adenoma, n (‰)	209 (5.3)	-
Advanced adenoma, n (‰)	58 (1.5)	-
**Screening finding after the age of 50**		
FIT positive, n (%)	4,143 (10.5)	27,460 (12.3)
All adenoma, n (‰)	845 (21.5)	6,679 (29.8)
Advanced adenoma, n (‰)	358 (9.1)	3,151 (14.1)

© 2025 Chiu HM et al. *JAMA Oncology*.

eTable 8. Efficacy of Early Screening vs Regular Screening for Colorectal Cancer Incidence and Mortality Using 3 Propensity Score–Based Adjustments

Propensity-Score-based Adjustments	Colorectal Cancer Incidence		Colorectal Cancer Mortality	
	aRR	(95% CI)	aRR	(95% CI)
**Matching**	0.79	(0.67, 0.94)	0.61	(0.38, 0.98)
**Continuous**	0.77	(0.66, 0.91)	0.58	(0.38,0.91)
**Decile**	0.77	(0.65, 0.90)	0.58	(0.37, 0.89)

© 2025 Chiu HM et al. *JAMA Oncology*.

eTable 9. Results of the Efficacy of the Early Screening Group vs the Regular Screening Group With the Fully Adjusted Model

	Colorectal cancer incidence		Colorectal cancer death	
	Crude RR (95% CI)	Adjusted RR (95% CI)	Crude RR (95% CI)	Adjusted RR (95% CI)
**Early Screening versus Regular Screening**				
40-49 vs after 50	0.61 (0.52-0.72)	0.78 (0.67-0.91)	0.44 (0.28-0.67)	0.61 (0.39-0.95)
**Sex**				
Male vs. Female	1.49 (1.05-1.36)	1.33 (1.21-1.46)	1.43 (1.14-1.79)	1.19 (0.95-1.49)
**Birth cohort**				
1952-1959 vs. 1960-1966	1.87 (1.69-2.07)	2.17 (1.96-2.41)	2.18 (1.69-2.81)	2.76 (2.13-3.58)
**Family history***				
Yes vs. No	1.33 (1.11-1.60)	1.37 (1.14-1.65)	1.53 (1.00-2.34)	1.59 (1.04-2.43)
**Number of screening rounds with the uptake after 50**				
2 or more vs. 1	0.54 (0.49-0.59)	0.48 (0.43-0.53)	0.33 (0.26-0.43)	0.29 (0.22-0.37)

* Only individuals with colorectal cancer in their first-degree relatives are considered positive for family history in this study

© 2025 Chiu HM et al. *JAMA Oncology*.

eFigure 1. Cumulative Incidence of Colorectal Cancer in Male and Female Population Between the Early and Regular Screening Groups

(A) Male

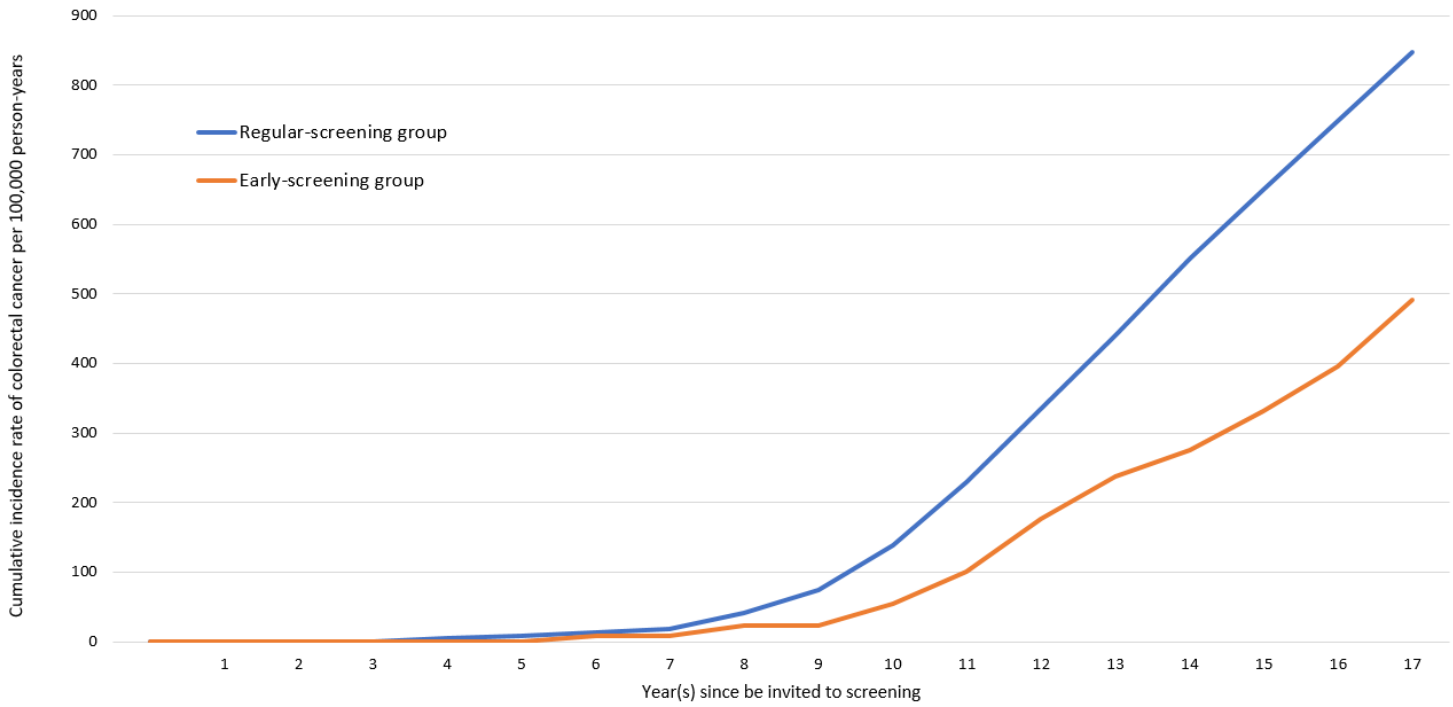

Number of person at risk

	1	2	3	4	5	6	7	8	9	10	11	12	13	14	15	16	17
Early-screening group	13,026	13,026	13,026	13,026	13,026	13,026	13,025	13,025	13,023	13,023	13,019	13,013	13,003	12,995	12,817	12,298	11,463
Regular-screening group	103,303	103,303	103,303	103,303	103,297	103,294	103,289	103,284	103,261	103,227	103,160	103,066	102,958	102,847	101,386	97,865	92,861

© 2025 Chiu HM et al. *JAMA Oncology*.

(B) Female

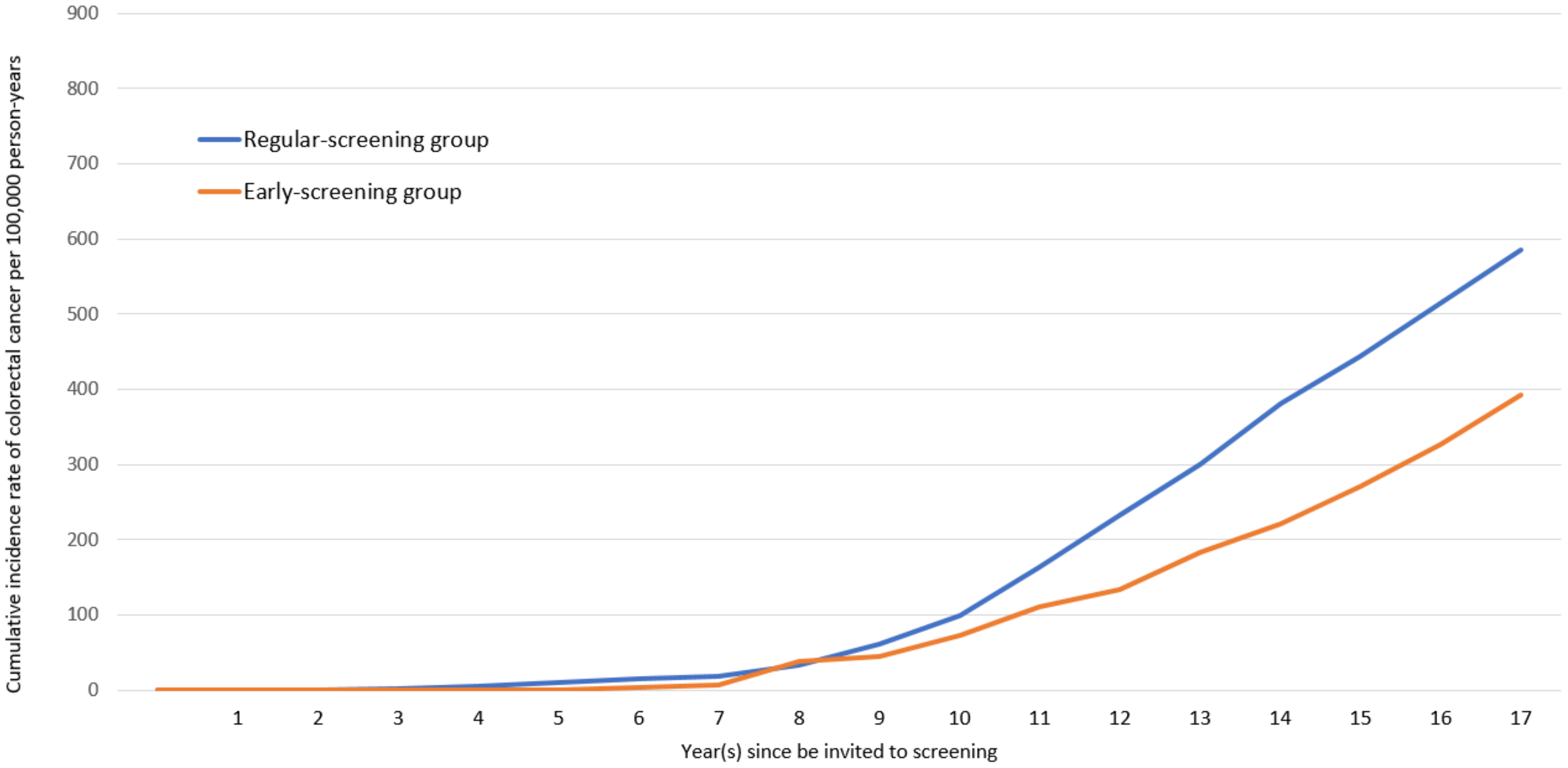

Number of person at risk

Early-screening group	26,289	26,289	26,289	26,289	26,289	26,289	26,288	26,287	26,279	26,277	26,270	26,260	26,254	26,241	25,877	24,810	23,165
Regular-screening group	120,507	120,507	120,506	120,504	120,500	120,494	120,488	120,484	120,467	120,434	120,386	120,310	120,228	120,144	118,054	113,063	106,328

© 2025 Chiu HM et al. *JAMA Oncology*.

eFigure 2. Cumulative Mortality of Colorectal Cancer in Male and Female Population Between the Early and Regular Screening Groups

(A) Male

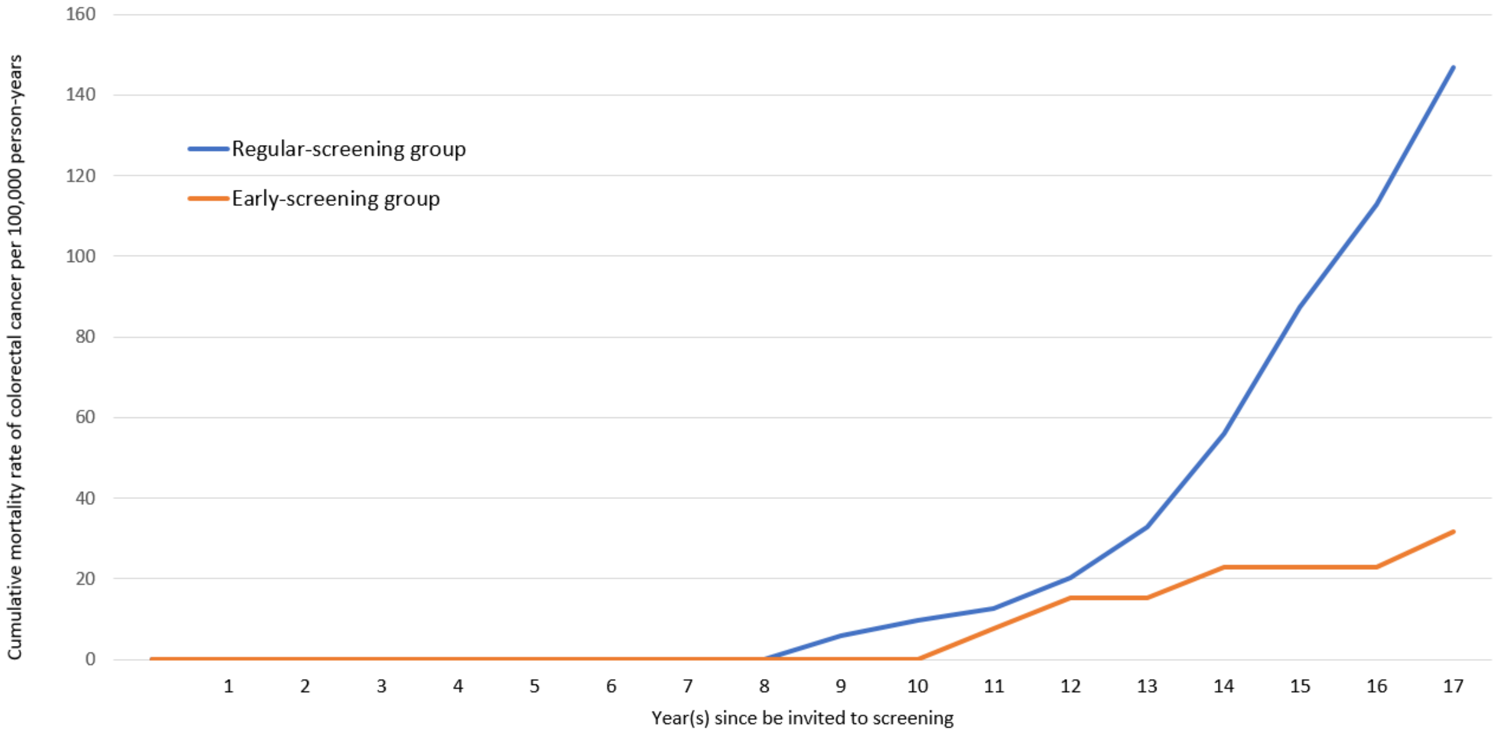

Number of person at risk

	1	2	3	4	5	6	7	8	9	10	11	12	13	14	15	16	17
Early-screening group	13,026	13,026	13,026	13,026	13,026	13,026	13,026	13,026	13,026	13,026	13,026	13,025	13,024	13,024	12,848	12,335	11,508
Regular-screening group	103,303	103,303	103,303	103,303	103,303	103,303	103,303	103,303	103,303	103,297	103,293	103,290	103,282	103,269	101,893	98,433	93,484

© 2025 Chiu HM et al. *JAMA Oncology*.

(B) Female

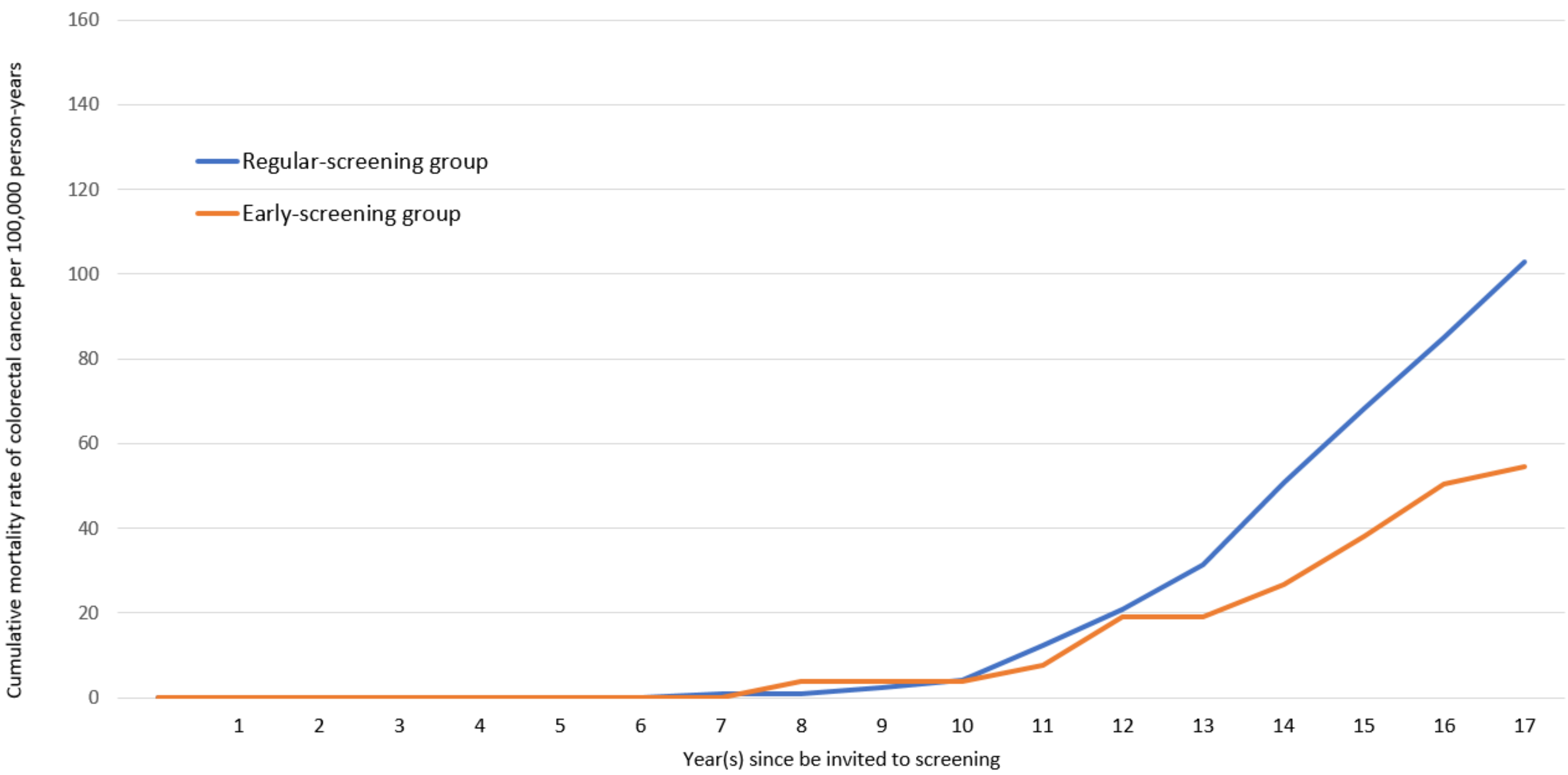

Number of person at risk

Early-screening group	26,289	26,289	26,289	26,289	26,289	26,289	26,289	26,289	26,288	26,288	26,288	26,287	26,284	26,284	25,926	24,867	23,228
Regular-screening group	120,507	120,507	120,507	120,507	120,507	120,507	120,507	120,506	120,506	120,504	120,502	120,492	120,482	120,469	118,449	113,500	106,807

© 2025 Chiu HM et al. *JAMA Oncology*.

eFigure 3. Cumulative Incidence and Mortality of Colorectal Cancer by the Participation Status of Screening at Ages Between 40 and 49 Years

(A) Cumulative incidence

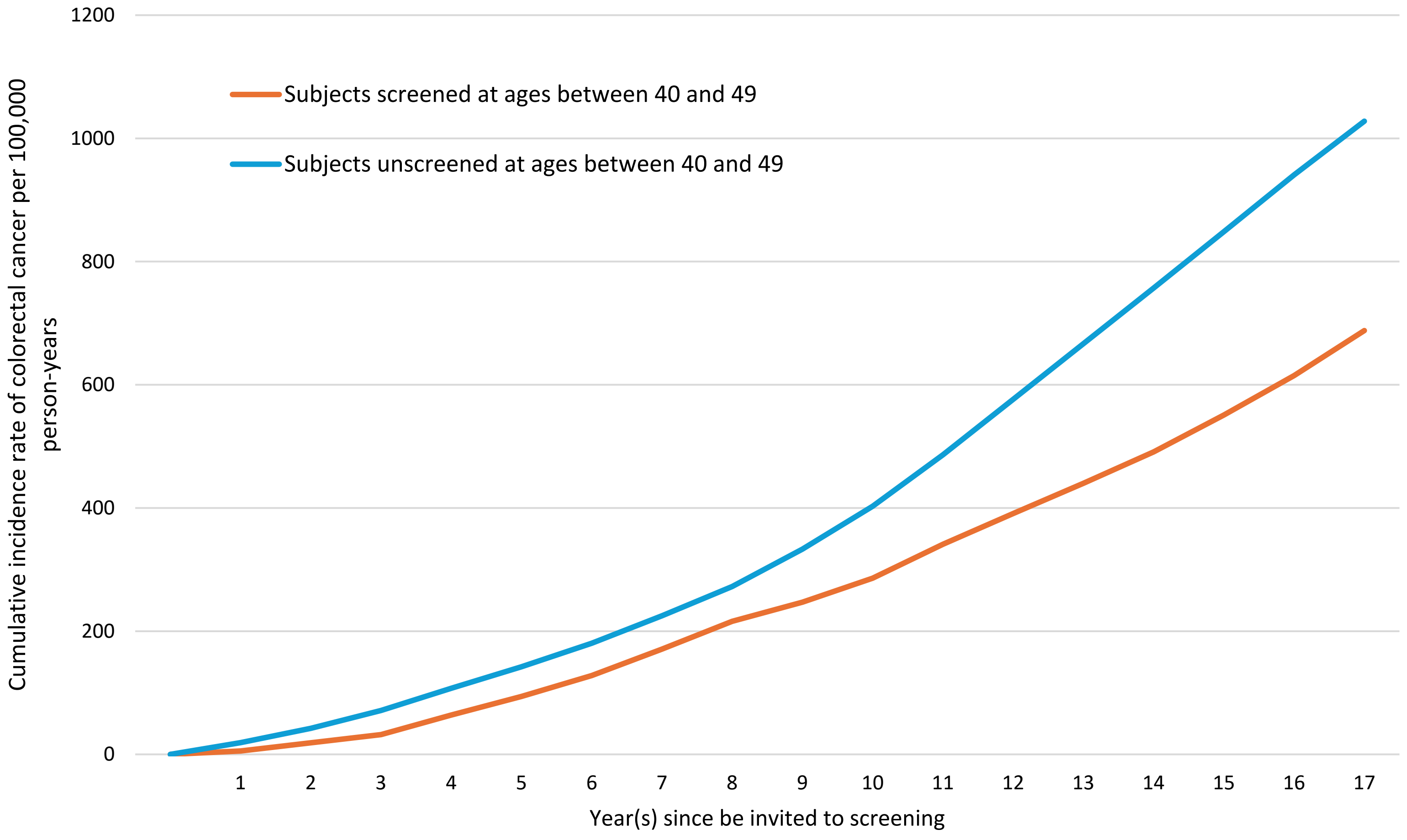

© 2025 Chiu HM et al. *JAMA Oncology*.

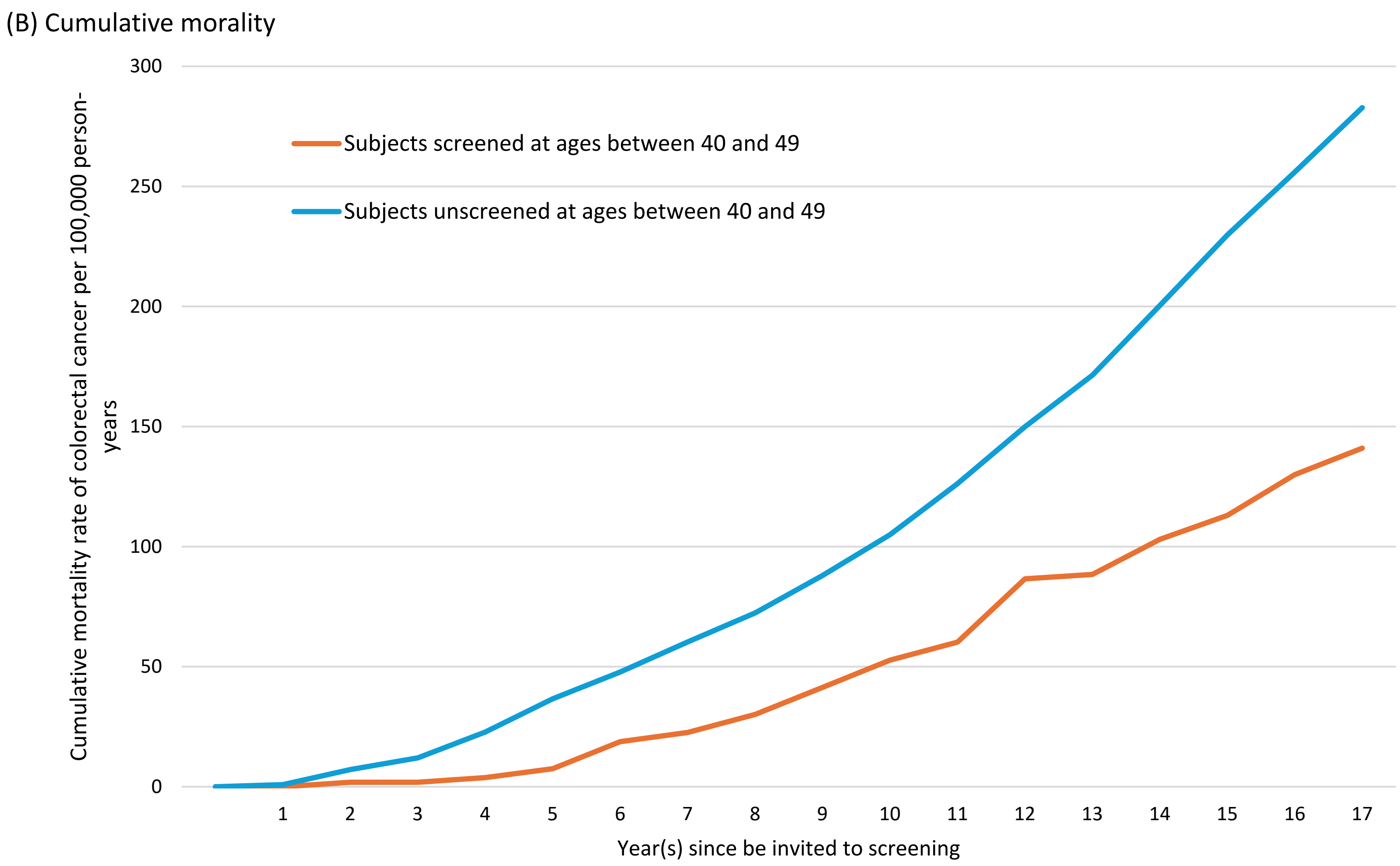

© 2025 Chiu HM et al. *JAMA Oncology*.

eFigure 4. Cumulative Incidence and Mortality of Colorectal Cancer by the Participation Status of Screening by Age

(A) Cumulative incidence

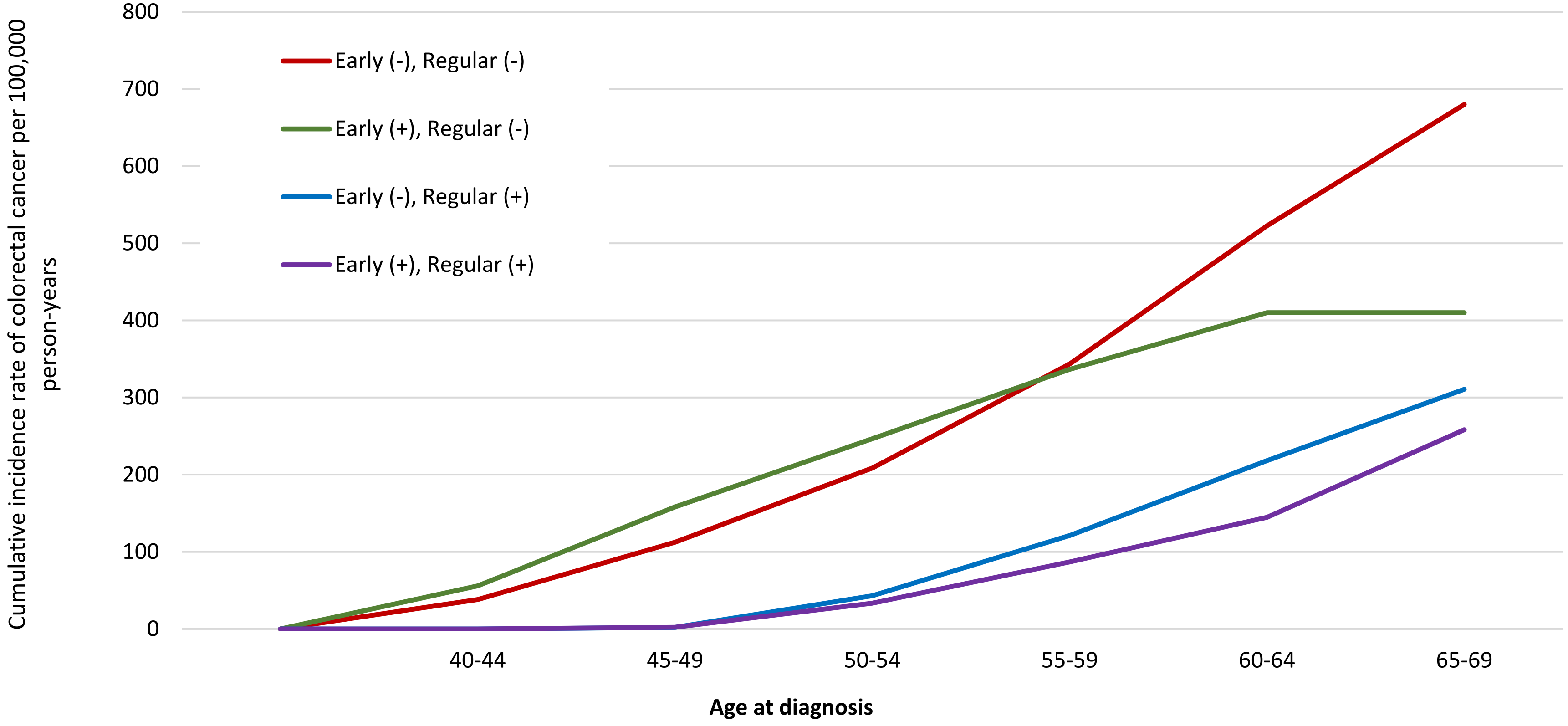

© 2025 Chiu HM et al. *JAMA Oncology*.

(B) Cumulative mortality

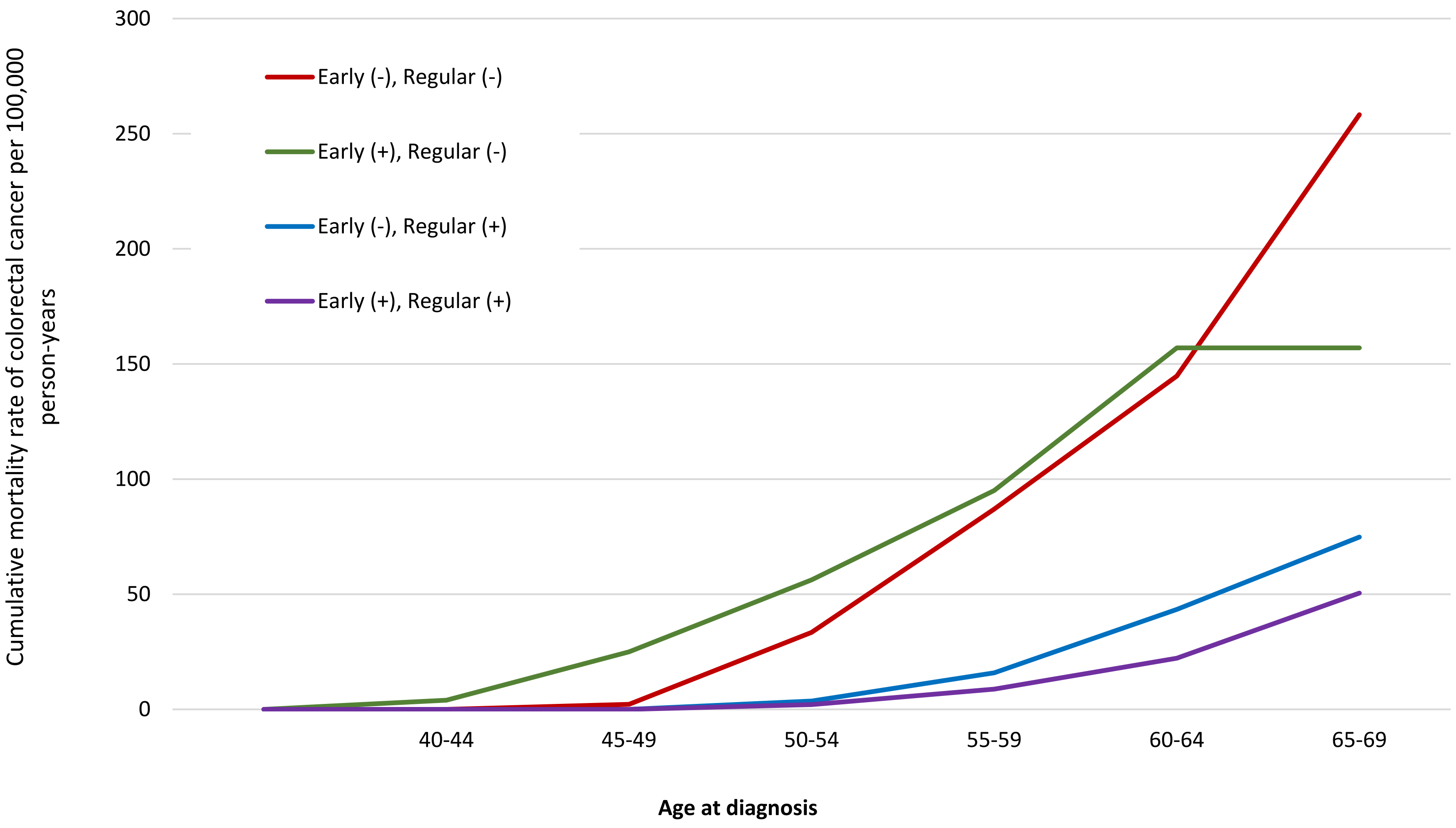

© 2025 Chiu HM et al. *JAMA Oncology*.
