# Supplementary material for: Long-Term Effectiveness Associated With Fecal Immunochemical Testing for Early-Age Screening
Source: JAMA Oncol. 2025 Jun 12;11(8):846–54. doi: 10.1001/jamaoncol.2025.1433 (PMC12163714; doi:10.1001/jamaoncol.2025.1433)
Supplement: Supplement 2. — Data Sharing Statement [file jamaoncol-e251433-s002.pdf]

Data Sharing Statement

Chiu. Long-Term Effectiveness Associated With Fecal Immunochemical Testing for Early-Age Screening. *JAMA Oncol*. Published June 12, 2025. doi:10.1001/jamaoncol.2025.1433

Data
Data available: No
